# Effects of botulinum toxin A and/or bimanual task-oriented therapy on upper extremity activities in unilateral Cerebral Palsy: a clinical trial

**DOI:** 10.1186/s12883-015-0404-3

**Published:** 2015-08-19

**Authors:** Lucianne Speth, Yvonne Janssen-Potten, Eugene Rameckers, Anke Defesche, Bjorn Winkens, Jules Becher, Rob Smeets, Hans Vles

**Affiliations:** Adelante, Paediatric Rehabilitation, Onderstestraat 29, 6301 KA Valkenburg, The Netherlands; Adelante, Centre of Expertise in Rehabilitation and Audiology, Zandbergsweg 111, 6432 CC Hoensbroek, The Netherlands; Department of Rehabilitation Medicine, Maastricht University, Research School CAPHRI, PO Box 616, 6200 MD Maastricht, The Netherlands; Department of Methodology and Statistics, Maastricht University, Research School CAPHRI, Maastricht, The Netherlands; Department of Rehabilitation Medicine, Free University Medical Centre, De Boelelaan 1118, 1081 HZ Amsterdam, The Netherlands; Department of Rehabilitation Medicine, Maastricht University Medical Centre, PO Box 5200, 6202 AZ Maastricht, The Netherlands; Department of Neurology, Maastricht University Medical Centre, Maastricht, The Netherlands; Department of Neurology, Maastricht University, Research School GROW, Maastricht, The Netherlands

## Abstract

**Background:**

This study reports on the effects of botulinum toxin A (BoNT-A) injections in the upper extremity (UE) in children with unilateral Cerebral Palsy (uCP) combined with bimanual task-oriented therapy (BITT) or either treatment modality performed separately. Bimanual activities were measured with the Assisting Hand Assessment (AHA), the ABILHand-Kids questionnaire (AK), the Observational Skills Assessment Score (OSAS). Goal achievement was measured with Goal Attainment Scaling (GAS), using blind video assessment, and the Canadian Occupational Performance Measure (COPM).

**Methods:**

Thirty-five children, mean age 7.14 years (SD 2.63), 11 Manual Ability Classification Score (MACS) I, 15 MACS II and 9 MACS III, participated. The trial started with four study groups: BoNT-A-only (*n* = 5), BITT-only (*n* = 11), BoNT-A + BITT (*n* = 13), and control (*n* = 6). Twenty-two children were randomised, 13 children received their parents’ preferred treatment: BoNT-A + BITT or BITT-only. Three comparisons were analysed: BITT (BoNT-A + BITT and BITT-only; *n* = 24) versus no BITT (BoNT-A-only and control; *n* = 11), BoNT-A (BoNT-A-only and BoNT-A + BITT; *n* = 18) versus no BoNT-A (BITT-only and control; *n* = 17), and the additional effect of BoNT-A (BoNT-A + BITT versus BITT-only). Follow-up time: 24 weeks.

**Results:**

No significant differences between the groups were found on the AHA. The amount of use of both hands on the OSAS was significantly better in the BoNT-A group in the beading and sandwich-making task. The BoNT-A group also showed significant improvement in the quality scores of the OSAS: the wrist position during grasping and holding, especially in the younger children. The BITT group improved significantly on the AK and significantly more on the performance and satisfaction scores of the COPM at 12 and 24 weeks regarding several goals. BoNT-A showed a significant negative effect at 12 and 24 weeks in the most important goal. BITT, more than BoNT-A + BITT, showed positive effects on the GAS score at 12 (significant), 18 and 24 weeks.

**Conclusions:**

BoNT-A has a positive effect on quality of movement and amount of use of the affected UE during the 3 months’ working time. BoNT-A has no additional effect on bimanual performance and goal achievement. BITT has a positive effect on goal achievement and bimanual performance, even up to 6 weeks after therapy had stopped.

Trial registration: Current Controlled Trials ISRCTN69541857.

**Electronic supplementary material:**

The online version of this article (doi:10.1186/s12883-015-0404-3) contains supplementary material, which is available to authorized users.

## Background

According to the Cochrane review [1], the combination of botulinum toxin A (BoNT-A) in the upper extremity (UE) and intensive physiotherapy(PT)/occupational therapy(OT) is more effective than intensive therapy alone in improving the activity level of the International Classification of Functioning (ICF, www.who.int/entity/classifications/icf/en/) and in goal achievement in children with unilateral spastic Cerebral Palsy (uCP). Assessments used to measure effects at the activity level in the studies in this review are the Melbourne Assessment of Unilateral Upper Limb Function (MUUL) [2], the Quality of Upper Extremities Skills Test (QUEST) [3] and the Pediatric Disability Inventory (PEDI) [4]. Goal achievement was measured with Goal Attainment Scaling (GAS) [5] and the Canadian Occupational Performance Measure (COPM) [6].

Although the studies themselves reported no significant differences on the MUUL and limited effects on the QUEST, a meta analysis of the pooled data in this review [1] showed slight positive effects on both tests 3 months after injection of BoNT-A, which had disappeared at 6 months. On the PEDI functional skills score almost no effect was found. There were clear positive effects on GAS and limited positive effects on the COPM concluded. Olesch et al. studied young children (mean age 3 years 8 months) with uCP and found no significant differences on the QUEST between repeated BoNT-A + OT and OT alone. However, they did find positive effects on GAS of BoNT-A + OT [7]. The way GAS was performed in these studies (scoring by parents or therapists) had subjective elements. This should be taken into account, while interpreting these results.

Also, in a more recent review evaluating upper limb therapies for uCP there was a modest supplementary effect of BoNT-A as an adjunct to OT to improve unilateral capacity, quality of movement, with the MUUL and the QUEST, and a clear positive effect on achieving individualised treatment outcomes [8]. In this review in only one study looking at the effect of BoNT-A on strength in children receiving resistance training, the assisting hand assessment (AHA) was used to measure bimanual performance [9].

OT after BoNT-A, modified constraint induced movement therapy (mCIMT), bimanual intensive therapy (BIMT) and goal-directed and context-focused therapies are considered to be evidence-based effective improving UE activities [8, 10]. After BoNT-A, mCIMT and bimanual OT (BOT) each consisting of 16 one-hour therapy sessions during 6 weeks both with home therapy elements, were equally effective in young children with uCP, although in hindsight the home programme intensity in the BOT group was significantly less [11]. In this study and in several studies reporting effects of CIMT and/or BIMT, they used the AHA, a reliable, responsive tool to measure the effective use of the assisting hand in bimanual performance [12, 13]. With the AHA, in several studies comparing the effects of two forms of intensive therapy currently often used in uCP, CIMT and BIMT, no difference between these treatments could be demonstrated [14–16].

At the start of our study, there were no studies measuring the effect of (additional) BoNT-A on bimanual performance. Because we considered this effect, the effective use of the affected hand in bimanual play and self-care, of utmost importance, we chose the AHA as main outcome measurement in this study.

BoNT-A has a clear effect on tone reduction [1, 17], influencing movement fluency. As fluency corresponds with quality of use of the affected hand, measuring this aspect, i.e., the capacity of the affected hand as an assisting hand in bimanual activities, is necessary in our opinion. For this, the Observational Skills Assessment Score (OSAS) was developed and tested for its clinimetric properties. It appeared to be an appropriate instrument [18].

Therefore, in the present study, we investigated the effects of BoNT-A injections in the UE combined with bimanual task-oriented therapy (BITT) or either treatment modality performed separately on the ICF activity level, on bimanual performance, measured with the AHA and the ABILHAND-Kids questionnaire (AK) [19], on the amount of use and capacity of the assisting hand with the OSAS, and on goal achieving, measured with GAS (scored blindly on video recordings) and the COPM.

## Methods

### Design

This study, called BoBiVa (Botuline toxine Bimanuele Vaardigheden), was designed as a multicentre randomised controlled trial on the effect of BoNT-A injections combined with bimanual task-oriented therapy (BITT), or either separately, in children with uCP on UE functions and skills. The trial was registered at the ISRCTN site: http://www.controlled-trials.com/ISRCTN69541857. The METC Atrium-Orbis-Zuyd (ref: 06-p-33) and the Dutch CCMO (ref: NL12005.096.06) provided medical ethics approval. The parents gave their informed consent for their children to participate in the measurements and therapies of this study. Initially, besides Adelante/Maastricht University Medical Centre, two other centres, VUMC in Amsterdam and UMC St. Radboud in Nijmegen, participated in this trial. Here, the BoNT-A injections were given. Due to disappointing patient enrolment, other centres, in which the BITT programme was performed, joined later. These recruitment problems also led to a change in study design: with approval of the METC this became a clinical study, as is further mentioned in the ‘Randomisation’ section. In this paper, the results at the ICF-activity level are reported. Originally we wanted to use a factorial design to determine the effects of BoNT-A, BITT, and the additional effect of BoNT-A to BITT (Table 1). As far as possible, the CONSORT guidelines for reporting parallel group randomised trials were followed [20].

**Table 1:** Factorial study design

	BoNT-A yes	BoNT-A no	
BITT yes	BoNT-A + BITT (*n* = 13)	BITT-only (*n* = 11)	24
BITT no	BoNT-A-only (*n* = 5)	Control (*n* = 6)	11
	18	17	

*BoNT-A* botulinum toxin A. *BITT* bimanual task-oriented therapy

### Participants

Children with spastic uCP, aged 2.5 to 12 years, Manual Ability Classification System (MACS) [21] levels I-III, Zancolli hand impairment grade I to IIB [22], who were mentally able to perform the measurements and attend the therapy programme, were considered eligible for inclusion. The study excluded children without active hand function (Zancolli III), severe structural contractures (elbow extension deficit more than 20° and/or supination deficit more than 45° and/or wrist extension deficit more than 30°) and children who had undergone a treatment of the affected hand (BoNT-A injections less than 9 months before, or hand surgery).

### Therapy programme

The BITT programme consisted of half an hour of PT and one hour of OT, 2 times a week, for 12 weeks. Children in the outpatient clinic had the PT and OT (1 ½ h) after each other, 2 times a week. Children who were at the special school had the therapy during school days. At the start, bimanual goals were set using the COPM [6]. Examples of goals that were being targeted in the therapy are: pulling up trousers and closing the button, cutting paper with scissors, pulling apart LEGO bricks, tying shoelaces, closing the zip of a jacket. These goal-directed activities were practiced as much as possible in the child’s relevant context. The programme was individually tailored to the child based on a task analysis of the selected goals performed at the start of the treatment and was based on principles of motor learning, strength training and/or improving range of motion (ROM). When weakness was the main problem in closing the button of a pair of jeans, this was trained by asking the child to pull up his trousers and then close the button several times, starting with wider trousers and an easier clasp. When ROM was a problem and the finger flexors were shortened, the children had to wear a finger flexor stretching night splint. Also day splints facilitating hand function were used. Additionally, parents were asked to stimulate their child to practice these goal-directed dressing and playing activities every day in a home-exercise programme. They practiced these activities several times a day during grooming and while playing. Parents reported that during the day they spent 40 to 60 min practising these goals (56–84 h of exercise in total). All therapists of the participating centres were trained in this special goal-directed, task-specific bimanual therapy. ER and AD trained the therapists to perform the BITT programme during a 6 h training course. During the BITT programme, these therapists were coached by AD. Feedback was given on their task analysis using video recordings of the child performing his/her individual goal. The children in the BoNT-A-only and control group continued their usual therapy with a maximum frequency of 1 h a week. We requested their therapists not to work at improving bimanual goals. The physiotherapists of the children in these treatment groups were unaware of the goal setting.

### BoNT-A injection technique and dosage

The most spastic muscles hampering function and bimanual activities were injected once in both the BoNT-A treatment groups; in the BoNT-A + BITT group, the BoNT-A was injected 4 days to a maximum of 2 weeks before the start of the BITT programme. Based on a video of the child performing his or her most important goal and on UE spasticity measurements, the three physicians of the initial participating centres decided during teleconferencing which muscle had to be treated and in what dose. Dysport® (Ipsen) was used with dilution 25 U/0.1 ml and with a dose 3 times the dose of Botox® (Allergan), 6–9 U/kg body weight for muscles above the elbow and 3–6 U/kg body weight for muscles in the forearm, limited to no more than 150 units (0.6 ml) at one injection site. In the intrinsic thumb muscles, the maximum dose was 50 U per muscle; mostly 25 U per muscle was injected. The total maximum Dysport® dose was 1000 U per child per session [23]. The injections took place under general anaesthesia in the day care department of the three initially participating centres. The muscles that had to be injected were located by electro-stimulation; the BoNT-A was injected with a special Teflon® coated needle. The most frequently injected muscles were the adductor pollicis, flexor carpi ulnaris and radialis and pronator teres. For dose and which muscle was injected see our publication of the effect of BoNT-A and/or BITT on upper extremity functions [17], or Additional file 1.

### Outcome measures

Two baseline measurement sessions with a maximum of 2 weeks in between, and four follow up sessions at 6, 12, 18 and 24 weeks after BoNT-A/start BITT were performed. The AHA, the primary outcome measure, was administered at the second baseline session and at 12 and 24 weeks. The AK was taken at the first baseline session and all follow-up sessions. Both the AHA (a video assessment) and the AK (a questionnaire) were performed to measure effectiveness of use of the affected hand (AH) in bimanual performance. To measure amount and quality of use of the AH i.e., capacity, the OSAS [18] was administered. The AHA and the OSAS were administered by a certified, experienced occupational therapist (OTst). With the COPM, which was administered at the first baseline session, 3 goals in which bimanual hand use was needed were determined. Evaluation of these goals with the COPM took place at 12 and 24 weeks. The most important problem that emerged from the COPM was video recorded at the second baseline measurement session. Watching this video, goal attainment scaling (GAS) was performed by the first author (LS) and an experienced OTst (AD). A 6-point scale was used with 0 representing the expected level of success, 2 clearly more than expected and −3 worse than before [5]. At all the other measurement sessions, new video recordings of this main goal were made. Afterwards these GAS videotapes and those of the AHA and OSAS were renamed and scored by trained assessors (PTsts and OTsts). These assessors were not involved in the treatment and unaware at which time session the video was taken, so blinding was guaranteed. The AHA score was converted to the AHA 0–100 units score. Additionally, measurements at function level of the ICF were performed. These results have been reported earlier [17].

### Sample size

Power analysis was done on the AHA, as this was the main outcome measurement of the BoBiVa study at the ICF activity level. At the start of this study, the study of Eliasson [24] was the only known study that used the AHA as an evaluative measurement. We assumed that the difference between groups (mCIMT and control) as found by Eliasson (mean change for the treatment group 1.23 (SD 1.04) and for the control group 0.24 (SD 0.85)) was the minimum clinically relevant difference to be detected in our study. Using the same variation, a two-sided alpha of 0.05 and a beta of 0.10 (i.e., power of 90 %), a minimum of 50 children in total were needed to detect the main effects of the treatment comparisons (effect of BoNT-A, BITT or BoNT-A + BITT) on daily hand use.

### Randomisation

The trial started with a factorial design in 2008. There were 4 study groups: 1) BoNT-A-only, 2) BITT-only, 3) BoNT-A + BITT and 4) control group (Table 1). To improve comparability of the groups, randomisation into the four treatment groups took place after pre-stratification for centre and age group: 2.5–6 and 7–12 years. Randomisation was done after the two baseline measurements and before start of the treatment. Using opaque envelopes ensured allocation concealment. An independent researcher generated random allocation sequence. LS and JB enrolled participants. YJ assigned participants to the intervention by handing out the opaque envelopes. Because of recruitment problems, we abandoned the randomisation into 4 groups after official approval of the medical ethics committee. This adaptation was necessary because many parents did not want their child to participate, because they either had a strong preference for or aversion to BoNT-A treatment. Also, they considered the risk of allocation of their child to either the control or the BoNT-A-only group to be too high. From March 2010 on, the children were only allocated to the BoNT-A + BITT or the BITT-only group. The scientific importance of random allocation was discussed with the parents. If the parents agreed, the children were randomised; otherwise, their child was allocated to the group of their preference. As this was the choice of the parents and not of a physician, we do not expect that it has led to confounding by indication.

### Statistical analysis

Categorical and numerical variables were presented by number of patients (%) and by means (SD), respectively. The longitudinal effects of bimanual therapy (BITT versus no-BITT) and BoNT-A (BoNT-A versus no-BoNT-A) on the numerical outcome measures were assessed using linear mixed models to correct for baseline differences and to account for the correlations between repeated measures within the same patient and for missing data by using likelihood methods, which assume the data to be missing at random. The fixed part of the models included BITT (yes/no), BoNT-A (yes/no), time (0, 6, 12, 18 and 24 weeks), BITT*time, and BoNT-A*time. Restricted maximum likelihood estimation was used and the covariance structure for the repeated measures (compound symmetry (CS), heterogeneous CS, first-order autoregressive (AR1), heterogeneous AR1, Toeplitz (TP), heterogeneous TP or unstructured) was selected based on Akaike’s information criterion. As for the additional effect of BoNT-A (BoNT-A + BITT versus BITT-only) on the outcome measures, a similar linear mixed model (without the variables BITT and BITT*time) was applied to the data of the patients who received either BITT-only or BoNT-A + BITT. For the ordinal GAS score, the differences between the groups were assessed using the Kruskal-Wallis test. A two-sided p-value ≤ 0.05 was considered statistically significant. All analyses were performed using IBM SPSS Statistics for Windows (Version 20.0. Armonk, NY: IBM Corp).

## Results

Patients were enrolled from January 2008 until December 2010. The last follow-up measurement took place in September 2011. Initially, thirty-six children with uCP were enrolled in the study. One child, who was allocated to the BITT-only group, withdrew after the baseline measurements because the participating centre had problems organizing BITT. Therefore, a total of 35 children participated in the study. Twenty-four were born at full term, 6 at 37 weeks and 5 were born prematurely (25–35 weeks gestation).

**Fig. 1:**
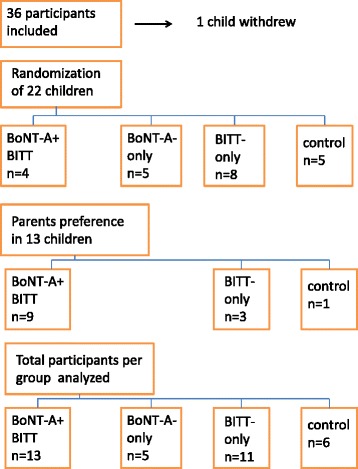
Allocation flow diagram participants after 2 baseline measurements

Twenty-two children were allocated by randomisation; in 13 children the parents’ preference was followed (Fig. 1) of whom nine were allocated to the BoNT-A + BITT group and four to the BITT-only group. The parents of one young child in the latter group indicated after the baseline measurements that they were not able to bring their child for therapy twice a week. They agreed to let him perform the measurements only, so the child was allocated to the control group. Two children who were randomly assigned to the control group missed the outcome measurements of week 6, 18 and 24. One because she underwent surgery to improve walking; the parents of the other child were no longer willing to cooperate with the measurements. Their baseline and 12 week measurement results were analysed.

We used a registration form for Serious Adverse Events (SAE’s) and Suspected Unexpected Serious Adverse Reactions (SUSAR’s), but no adverse reactions were reported.

In Table 2, characteristics and outcome measures at baseline of all children and treatment groups are depicted. The children in the control group and less in the BoNT-A-only group were clearly younger. Hand function impairment (Zancolli grade) was worst in the control group and in the BoNT-A-only group bimanual performance i.e., the MACS was worst. Children in the control group scored relatively low on the AHA units and the ABILHAND-Kids logit units’ score. Overall, there were no significant differences between the groups at baseline.

**Table 2:** Characteristics and outcome measures at baseline total group of children

All children	BoNT-A + BITT	BoNT-A-only	BITT-only	Control	All
	*n* = 13	*n* = 5	*n* = 11	*n* = 6	*n* = 35
Age (years) mean (sd)	7.8 (2.7)	6.6 (3.5)	7.4 (2.4)	5.7 (2.0)	7.1 (2.6)
Hemi side					
Hemi right n (%)	9 (69.2)	3 (60.0)	6 (54.5)	1 (16.7)	19 (54.3)
Hemi left n (%)	4 (30.8)	2 (40.0)	5 (45.5)	5 (83.3)	16 (45.7)
Zancolli grade					
Zancolli I n (%)	9 (69.1)	2 (40.0)	6 (54.5)	2 (33.3)	20 (57.1)
Zancolli II A n (%)	3 (23.1)	3 (60.0)	4 (36.4)	2 (33.3)	11 (31.4)
Zancolli II B n (%)	1 (7.7)	0 (0.0)	1 (9.1)	2 (33.3)	4 (11.4)
MACS					
MACS I n (%)	2 (15.4)	2 (40.0)	4 (36.4)	3 (50.0)	11 (31.4)
MACS II n (%)	7 (53.8)	1 (20.0)	4 (36.4)	3 (50.0)	15 (42.9)
MACS III n (%)	4 (30.8)	2 (40.0)	3 (27.3)	0 (0.0)	9 (25.7)
AHA					
Units (SD)	56.0 (9.0)	56.4 (12.1)	59.3 (12.6)	49.5 (10.3)	56.0 (10.9)
ABILHAND-Kids					
Logit units (sd)	1.205 (0.915)	1.393 (0.430)	1.418 (0.766)	0.839 (1.494)	1.236 (0.927)
COPM mean of 3 goals					
Performance mean (sd)	4.2 (1.5)	3.5 (1.2)	3.4 (1.3)	3.2 (1.4)	3.7 (1.4)
Satisfaction mean (sd)	4.9 (2.1)	4.9 (1.9)	4.1 (1.5)	3.9 (1.8)	4.5 (1.8)

Mean scores and standard deviations

Because the OSAS has different standardised tasks for two age groups, 2.5 to 6 and 7 to 12 years old, the baseline values for these two age groups are given separately in the online Additional files 2 and 3. The group sizes were small in general. The younger children were more equally distributed across the four treatment groups. With a mean age of 3 years, the two young children in the BoNT-A-only group were clearly younger than their peers in the other treatment groups. Their performance was clearly worse in using both hands in the OSAS tasks. The control group contained only two children from the older age group and one of them performed badly on the AHA, and showed a low percentage of use in the OSAS construction tasks.

In Tables 3 - 6, the results of three comparisons are presented: a) BITT (BoNT-A + BITT and BITT-only) versus no-BITT (BoNT-A-only and control), b) BoNT-A (BoNT-A-only and BoNT-A + BITT) versus no-BoNT-A (BITT-only and control), and c) the additional effect of BoNT-A, i.e., BoNT-A + BITT versus BITT-only. The most relevant results of these comparisons will be discussed per table.

**Table 3:** AHA units and ABILHand-Kids logits, estimated means (standard errors)

	Follow-up weeks	BITT			BoNT-A			BoNT-A additional effect		
		Yes (BoNT-A + BITT and BITT-only)	No (BoNT-A-only and control)	*p*-value	Yes (BoNT-A + BITT and BoNT-A-only)	No (BITT-only and control)	*p*-value	BoNT-A + BITT	BITT-only	*p*-value
		*n* = 24	*n* = 11		*n* = 18	*n* = 17		*n* = 13	*n* = 11	
AHA Units (SE) *p*-value	0	57.5 (2.2)	52.6 (3.3)		55.0 (2.7)	55.1 (2.7)		56.0 (3.0)	59.3 (3.3)	
	12	59.6 (2.2)	54.2 (3.3)	0.811	55.5 (2.7)	58.3 (2.7)	0.142	56.7 (3.0)	62.7 (3.3)	0.183
	24	58.8 (2.2)	56.8 (3.4)	0.135	57.5 (2.7)	57.8 (2.8)	0.895	58.1 (3.0)	59.0 (3.3)	0.251
	overall			0.192			0.274			0.054
ABILHand-Kids Logit units (SE) *p* -value	0	1.301 (0.25)	1.092 (0.37)		1.210 (0.31)	1.183 (0.31)		1.204 (0.33)	1.418 (0.36)	
	6	1.481 (0.25)	1.000 (0.39)	0.468	1.162 0.31)	1.319 (0.32)	0.589	1.238 (0.33)	1.772 (0.37)	0.457
	12	***2.264*** (0.25)	***1.173*** (0.37)	***0.041***	1.384 (0.31)	2.054 (0.31)	0.080	1.868 (0.33)	2.672 (0.36)	0.239
	18	***2.190*** (0.26)	***0.796*** (0.39)	***0.016***	1.297 (0.31)	1.689 (0.33)	0.341	1.859 (0.33)	2.580 (0.38)	0.340
	24	2.230 (0.25)	1.114 (0.40)	0.128	1.369 (0.31)	1.976 (0.33)	0.241	1.980 (0.33)	2.470 (0.36)	0.659
	overall			0.132			0.457			0.748

At baseline and 6, 12, 18, and 24 weeks post BoNT-A/start BITT. Significant values are in italics and bold

With the AHA, our primary outcome measurement, no significant differences between the groups could be demonstrated (Table 3). From baseline until 12 weeks, 6 children deteriorated in AHA unit score, all children had BoNT-A injections; those who had not showed no decline (see the online Additional file 1 with individual AHA unit scores). Comparing BoNT-A versus no-BoNT-A and BoNT-A + BITT versus BITT-only, the groups in which the children did not receive BoNT-A showed a clear improvement from 0 to 12 weeks, whereas the BoNT-A groups did not. The children who received BITT scored significantly better at the ABILHAND-Kids logit units’ score at 12 and 18 weeks (BITT from 1.301 to 2.264 and 2.190 and no-BITT from 1.092 to 1.173 and 0.796); and not significantly better at 24 weeks (BITT 2.230, no BITT 1.114). Comparing BoNT-A versus no-BoNT-A and BoNT-A + BITT versus BITT-only, the no-BoNT-A and the BITT-only groups showed a clear improvement in the AK logit units’ score, maximum at 12 weeks.

In the younger age group the percentage of use of both hands of the OSAS treading beads task increased from 69.7 % at baseline to 75.5 % at 18 and 73.1 % at 24 weeks in the BoNT-A group, which was significantly better than in the no-BoNT-A group (decrease from 83.2 % at baseline to 79.1 % at 18 and 76.4 % at 24 weeks) (Table 4). In the older age group, BoNT-A had a significant positive effect on the percentage of use of both hands at 6 weeks and at 24 weeks in the sandwich-making OSAS task (Table 5).

**Table 4:** Amount and Quality of use of grasp and hold wrist scores, tasks younger age group

	Follow-up weeks	BITT			BoNT-A			BoNT-A additional effect		
		Yes (BoNT-A + BITT and BITT-only)	No (BoNT-A-only and control)	*p*-value	Yes (BoNT-A + BITT and BoNT-A-only)	No (BITT-only and control)	*p*-value	BoNT-A + BITT	BITT-only	*p*-value
		*n* = 8	*n* = 6		*n* = 6	*n* = 8		*n* = 4	*n* = 4	
OSAS percentage of use both hands	0	80.6 (4.6)	72.4 (5.4)		69.7 (5.4)	83.2 (4.6)		76.7 (4.7)	84.5 (4.7)	
	6	78.6 (3.9)	68.3 (4.7)	0.662	66.7 (4.6)	80.1 (4.0)	0.980	73.1 (4.2)	84.1 (4.2)	0.580
	12	76.6 (6.7)	59.5 (7.9)	0.213	62.5 (7.9)	73.6 (6.7)	0.724	73.8 (5.8)	79.4 (5.8)	0.758
Treading beads *p* -value	18	81.7 (4.2)	72.9 (5.0)	0.922	***75.5*** (4.9)	***79.1*** (4.3)	***0.049***	81.8 (5.1)	81.5 (5.1)	0.216
	24	80.3 (3.5)	69.1 (4.3)	0.546	***73.1*** (4.1)	***76.4*** (3.6)	***0.041***	80.9 (2.8)	79.7 (2.8)	0.123
	overall			0.716			0.072			0.156
OSAS Quality grasp wrist	0	2.32 (0.26)	2.01 (0.31)		2.12 (0.31)	2.21 (0.260		2.16 (0.35)	2.49 (0.35)	
	6	2.43 (0.26)	2.51 (0.31)	0.219	***2.94*** (0.31)	***2.00*** (0.27)	***0.003***	2.60 (0.35)	2.26 (0.35)	0.114
Treading beads (1–5) SDD 0.65*p* -value	12	2.55 (0.26)	2.59 (0.31)	0.397	***3.05*** (0.31)	***2.10*** (0.26)	***0.020***	2.81 (0.35)	2.29 (0.35)	0.149
	18	2.36 (0.26)	2.32 (0.31)	0.437	2.56 (0.31)	2.12 (0.27)	0.128	2.48 (0.35)	2.25 (0.35)	0.280
	24	2.48 (0.26)	2.40 (0.32)	0.500	2.66 (0.31)	2.21 (0.27)	0.127	2.54 (0.35)	2.42 (0.35)	0.387
	overall			0.542			***0.028***			0.464
OSAS Quality grasp wrist	0	2.26 (0.27)	2.16 (0.32)		2.29 (0.32)	2.13 (0.27)		2.10 (0.38)	2.41 (0.38)	
	6	2.49 (0.27)	2.27 (0.32)	0.665	***2.98*** (0.32)	***1.79*** (0.27)	***0.001***	2.75 (0.38)	***2.23 (0.38)***	0.059
Pop-Onz (1–5) SDD 0.55 *p* -value	12	2.50 (0.27)	2.49 (0.32)	0.802	***3.10*** (0.32)	***1.89*** (0.27)	***0.000***	***3.01*** (0.38)	***2.00 (0.38)***	***0.028***
	18	2.33 (0.27)	2.69 (0.32)	0.164	***2.94*** (0.32)	***2.08*** (0.27)	***0.032***	2.51 (0.38)	2.16 (0.38)	0.175
	24	2.32 (0.27)	2.37 (0.32)	0.385	***2.61*** (0.32)	***2.08*** (0.27)	***0.043***	2.28 (0.38)	2.36 (0.38)	0.315
	overall			0.553			***0.011***			0.189
OSAS Quality hold wrist	0	2.44 (0.26)	2.23 ((0.31)		2.19 (0.31)	2.48 (0.26)		2.23 (0.34)	2.65 (0.34)	
Treading beads (1–5) SDD 0.71 *p* -value										
	6	2.44 (0.26)	2.62 (0.32)	0.218	***3.00*** (0.31)	***2.06*** (0.27)	***0.000***	***2.71*** (0.34)	***2.17*** (0.34)	***0.020***
	12	2.58 (0.26)	2.60 (0.31)	0.445	***2.86*** (0.31)	***2.31*** (0.26)	***0.006***	2.76 (0.34)	2.40 (0.34)	0.055
	18	2.53 (0.26)	2.39 (0.32)	0.822	***2.68*** (0.31)	***2.23*** (0.27)	***0.017***	***2.72*** (0.34)	***2.34*** (0.34)	***0.050***
	24	2.47 (0.26)	2.34 (0.32)	0.815	2.50 (0.31)	2.31 (0.27)	0.117	2.37 (0.34)	2.58 (0.34)	0.573
	overall			0.728			***0.003***			0.086
OSAS Quality hold wrist	0	2.35 (0.27)	2.11 (0.31)		2.16 (0.31)	2.30 (0.27)		2.07 (0.36)	2.63 (0.36)	
	6	2.68 (0.27)	2.37 (0.32)	0.829	***3.12*** (0.31)	***1.93*** (0.27)	***0.000***	***2.99*** (0.36)	***2.36*** (0.36)	***0.007***
Pop-Onz (1–5) SDD 0.87 *p* -value										
	12	2.62 (0.27)	2.45 (0.31)	0.781	***2.94*** (0.31)	***2.13*** (0.27)	***0.001***	***2.87*** (0.36)	***2.37*** (0.36)	***0.010***
	18	2.33 (0.27)	2.65 (0.32)	0.051	***2.77*** (0.31)	***2.21*** (0.27)	***0.014***	2.33 (0.36)	2.34 (0.36)	0.198
	24	2.34 (0.27)	2.24 (0.31)	0.605	2.37 (0.31)	2.20 (0.27)	0.271	2.13 (0.36)	2.55 (0.36)	0.735
	overall			0.215			***0.000***			***0.024***

Estimated means (standard error). Significant values are in italics and bold

**Table 5:** Amount and Quality of use of grasp and hold wrist scores, tasks older age group

	Follow-up weeks	BITT			BoNT-A			BoNT-A additional effect		
		Yes (BoNT-A + BITT and BITT-only)	No (BoNT-A-only and control)	*p*-value	Yes (BoNT-A + BITT and BoNT-A-only)	No (BITT-only and control)	*p*-value	BoNT-A + BITT	BITT-only	*p*-value
		*n* = 16	*n* = 5		*n* = 12	*n* = 9		*n* = 9	*n* = 7	
OSAS percentage of use both hands	0	77.3 (2.4)	81.0 (4.3)		78.2 (3.0)	80.1 (3.5)		76.2 (3.3)	78.5 (3.7)	
	6	77.7 (2.4)	74.4 (4.7)	0.193	***80.3*** (3.1)	***71.9*** (3.8)	***0.019***	***82.8*** **(3.3)**	***72.3*** **(3.7)**	***0.011***
	12	77.3 (2.4)	77.4 (4.3)	0.472	78.5 (3.0)	76.2 (3.5)	0.324	77.9 (3.3)	76.8 (3.7)	0.484
	18	76.8 (2.4)	79.3 (4.7)	0.822	78.7 (3.1)	77.5 (3.8)	0.469	78.2 (3.3)	75.2 (3.7)	0.275
Making a sandwich *p* -value										
	24	75.8 (2.4)	76.7 (4.7)	0.602	***79.6*** (3.1)	***72.8*** (3.8)	***0.050***	79.3 (3.3)	72.2 (3.7)	0.057
	overall			0.737			0.127			0.087
OSAS Quality grasp wrist	0	1.62 (0.19)	1.66 (0.34)		1.59 (0.24)	1.70 (0.27)		1.65 (0.25)	1.56 (0.28)	
Making a sandwich (1–5) SDD 0.55 *p* -value										
	6	1.71 (0.19)	1.83 (0.35)	0.732	1.79 (0.24)	1.75 (0.28)	0.434	1.82 (0.25)	1.56 (0.28)	0.471
	12	1.51 (0.19)	1.79 (0.34)	0.315	1.67 (0.24)	1.62 (0.27)	0.399	1.57 (0.25)	1.43 (0.28)	0.841
	18	1.66 (0.19)	1.91 (0.35)	0.423	1.76 (0.24)	1.81 (0.28)	0.756	1.70 (0.25)	1.61 (0.28)	0.983
	24	1.64 (0.19)	1.75 (0.35)	0.823	1.75 (0.24)	1.64 (0.28)	0.279	1.81 (0.250	1.45 (0.28)	0.265
	overall			0.846			0.814			0.746
OSAS Quality grasp wrist	0	1.71 (0.22)	1.77 (0.39)		1.82 (0.28)	1.66 (0.32)		1.93 (0.29)	1.45 (0.33)	
	6	2.02 (0.22)	2.35 (0.41)	0.435	2.50 (0.28)	1.87 (0.33)	0.081	2.38 (0.29)	1.65 (0.33)	0.362
Construction small (1–5) SDD 0.47 *p* -value										
	12	2.00 (0.22)	1.64 (0.39)	0.163	2.14 (0.28)	1.49 (0.32)	0.064	2.31 (0.29)	1.69 (0.33)	0.615
	18	1.88 (0.22)	1.89 (0.41)	0.872	2.05 (0.28)	1.73 (0.33)	0.554	2.07 (0.29)	1.69 (0.33)	0.719
	24	1.76 (0.22)	1.42 (0.41)	0.226	1.70 (0.28)	1.48 (0.33)	0.830	2.00 (0.30)	1.50 (0.33)	0.926
	overall			0.192			0.207			0.747
OSAS Quality grasp wrist	0	1.47 (0.17)	1.53 (0.31)		1.47 (0.22)	1.53 (0.25)		1.52 (0.24)	1.41 (0.27)	
	6	1.64 (0.18)	2.09 (0.32)	0.130	***2.12*** (0.22)	***1.61*** (0.26)	***0.010***	1.90 (0.24)	1.38 (0.27)	0.107
Construction large (1–5) SDD 0.81 *p* -value										
	12	1.68 (0.17)	1.78 (0.31)	0.861	1.91 (0.22)	1.56 (0.250	0.086	1.90 (0.24)	1.45 (0.27)	0.242
	18	1.72 (0.17)	1.48 (0.33)	0.455	1.63 (0.22)	1.58 (0.26)	0.716	1.77 (0.24)	1.65 (0.27)	0.957
	24	1.50 (0.17)	1.11 (0.33)	0.352	1.46 (0.22)	1.15 (0.26)	0.341	1.73 (0.24)	1.24 (0.270	0.442
	overall			0.084			***0.026***			0.169
OSAS Quality hold wrist	0	1.74 (0.19)	1.76 (0.34)		1.65 (0.24)	1.85 (0.27)		1.73 (0.25)	1.72 (0.28)	
	6	1.98 (0.19)	2.05 (0.36)	0.881	***2.17*** (0.24)	***1.86*** (0.29)	***0.053***	***2.27*** (0.25)	***1.64*** (0.28)	***0.044***
Making a sandwich (1–5) SDD 0.85 *p* -value										
	12	1.62 (0.19)	1.90 (0.34)	0.420	1.77 (0.24)	1.75 (0.27)	0.434	1.72 (0.25)	1.49 (0.28)	0.506
	18	1.71 (0.19)	2.09 (0.36)	0.237	1.80 (0.24)	2.01 (0.29)	0.964	1.70 (0.25)	1.69 (0.28)	0.992
	24	1.76 (0.19)	1.82 (0.35)	0.899	1.70 (0.24)	1.88 (0.28)	0.937	1.82 (0.25)	1.66 (0.28)	0.380
	overall			0.794			0.307			0.293
OSAS Quality hold wrist	0	1.86 (0.22)	1.97 (0.40)		1.88 (0.28)	1.95 (0.32)		1.93 (0.30)	1.76 ((0.34)	
	6	2.08 (0.22)	2.37 (0.42)	0.628	***2.54*** (0.28)	***1.90*** (0.34)	***0.021***	2.44 (0.30)	1.71 (0.34)	0.078
Construction small (1.5) SDD 0.81 *p* -value										
	12	1.96 (0.22)	1.81 (0.40)	0.461	***2.28*** (0.28)	***1.50*** (0.32)	***0.005***	2.34 (0.30)	1.58 (0.34)	0.065
	18	1.98 (0.22)	1.69 (0.42)	0.293	***2.17*** (0.28)	***1.50*** (0.34)	***0.017***	2.27 (0.30)	1.69 (0.34)	0.199
	24	1.84 (0.22)	1.74 (0.42)	0.576	1.97 (0.28)	1.61 (0.34)	0.166	2.12 (0.30)	1.54 (0.34)	0.202
	overall			0.576			***0.037***			0.351
OSAS Quality hold wrist	0	1.59 (0.18)	1.52 (0.32)		1.67 (0.22)	1.44 (0.26)		1.69 (0.26)	1.49 (0.29)	
	6	1.74 (0.18)	1.80 (0.34)	0.678	2.00 (0.23)	1.54 (0.28)	0.400	1.97 (0.26)	1.50 (0.30)	0.367
Construction large (1–5) SDD 0.58 *p* -value										
	12	1.62 (0.18)	1.67 (0.32)	0.699	1.92 (0.22)	1.36 (0.26)	0.202	1.86 (0.26)	1.39 (0.29)	0.368
	18	1.89 (0.18)	1.30 (0.34)	0.104	1.77 (0.23)	1.42 (0.27)	0.663	2.04 (0.26)	1.74 (0.29)	0.729
	24	1.49 (0.18)	1.36 (0.34)	0.845	1.52 (0.23)	1.32 (0.27)	0.892	1.65 (0.26)	1.31 (0.29)	0.646
	overall			0.260			0.603			0.874

Estimated means (standard errors). Significant values are in italics and bold

In Table 4, the OSAS quality of use scores of the affected, assisting hand in the younger age group are shown. There was clear improvement in the grasp wrist and the hold wrist score at 6, 12, 18 and in the Pop-Onz task also at 24 weeks and overall in the BoNT-A group, comparing BoNT-A versus no-BoNT-A, and a decrease in the no-BoNT-A group in the beading and the Pop-Onz tasks, resulting in significant differences between these groups. Comparing BoNT-A + BITT to BITT-only, there were also significant differences in these tasks favouring BoNT-A at 6 and 12 weeks in the Pop-Onz task.

Table 5 displays the OSAS quality of use scores of the older children. Comparing BoNT-A to no-BoNT-A and BoNT-A + BITT to BITT-only, the grasp wrist score was significantly better favouring BoNT-A in the large construction task at 6 weeks and the hold wrist score in the sandwich-making task at 6 weeks and in the small construction task at 6, 12 and 18 weeks and overall.

The grasp finger score of the sandwich-making task differed significantly at 6 weeks comparing BoNT-A to no-BoNT-A (1.18 to 1.88 versus 1.69 to 1.60, p 0.011) and comparing BoNT-A + BITT to BITT-only (1.78 to 1.84 versus 1.76 to 1.63, p 0.020) favouring BoNT-A.

Performance and satisfaction scores of the COPM are depicted in Table 6. In all three goals there was a significant difference in improvement of performance and satisfaction scores at 12 weeks and overall and in the second and third goal also at 24 week satisfaction scores in the BITT versus no-BITT comparison favouring BITT. In the first goal the no-BoNT-A (comparing BoNT-A versus no-BoNT-A) and the BITT-only group (BoNT-A + BITT versus BITT-only) improved significantly more.

**Table 6:** COPM performance and satisfaction scores

	Follow-up weeks	BITT			BoNT-A			BoNT-A additional effect		
		Yes (BoNT-A + BITT and BITT-only)	No (BoNT-A-only and control)	*p*-value	Yes (BoNT-A + BITT and BoNT-A-only)	No (BITT-only and control)	*p*-value	BoNT-A + BITT	BITT-only	*p*-value
		*n* = 24	*n* = 11		*n* = 18	*n* = 17		*n* = 13	*n* = 11	
COPM goal 1 performance *p* -value	0	3.7	3.1		3.8	3.1		4.2	3.2	
	12	***7.8***	***5.1***	***0.008***	***6.0***	***6.8***	***0.042***	7.5	8.1	0.069
	24	7.3	5.2	0.067	***5.8***	***6.7***	***0.044***	***6.8***	***7.8***	***0.024***
	overall			***0.024***			0.065			0.058
COPM goal 1 satisfaction *p* -value	0	4.4	4.0		4–7	3.7		4.8	4.0	
	12	***8.5***	***5.6***	***0.007***	***6.6***	***7.6***	***0.019***	***7.9***	***9.1***	***0.031***
	24	7.9	5.8	0.099	***6.3***	***7.4***	***0.023***	***7.0***	***8.8***	***0.020***
	overall			***0.024***			***0.036***			***0.042***
COPM goal 2 performance *p* -value	0	3.7	3.5		4.1	3.1		4.2	3.1	
	12	***7.7***	***4.8***	***0.000***	6.3	6.2	0.207	7.8	7.5	0.299
	24	7.5	5.9	0.101	7.2	6.2	0.970	8.0	6.9	0.954
	overall			***0.001***			0.318			0.458
COPM goal 2 satisfaction *p* -value	0	4.6	4.8		5.1	4.3		5.1	4.1	
	12	***8.3***	***5.0***	***0.000***	6.7	6.6	0.367	8.2	8.4	0.211
	24	***8.1***	***5.9***	***0.016***	7.3	6.7	0.753	8.2	8.1	0.351
	overall			***0.001***			0.430			0.389
COPM goal 3 performance *p* -value	0	4.0	3.5		3.7	3.7		4.0	4.0	
	12	***8.0***	***4.3***	***0.000***	5.8	6.4	0.320	7.9	8.0	0.918
	24	7.8	5.8	0.107	6.8	6.8	0.881	7.9	7.6	0.750
	overall			***0.000***			0.525			0.884
COPM goal 3 satisfaction *p* -value	0	4.5	4.6		4.9	4.2		4.7	4.3	
	12	***8.5***	***4.8***	***0.001***	6.2	7.0	0.137	8.2	8.7	0.361
	24	***8.0***	***5.2***	***0.002***	6.5	6.7	0.287	8.0	8.0	0.656
	overall			***0.003***			0.301			0.558

Estimated means. Significant values are in italics and bold

**Fig. 2:**
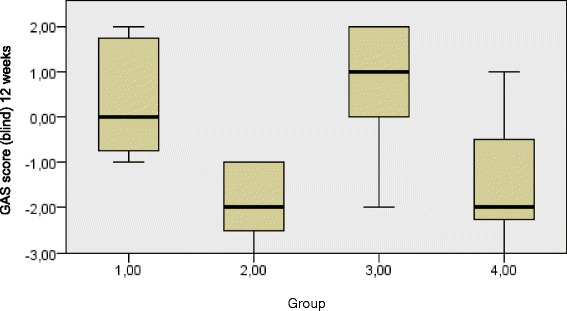
GAS blind score at 12 weeks medians and interquartile ranges per treatment group. Group 1 = BoNT-A + BITT, 2 = BoNT-A-only, 3 = BITT-only, 4 = control. Kruskal Wallis test p 0.002

**Fig. 3:**
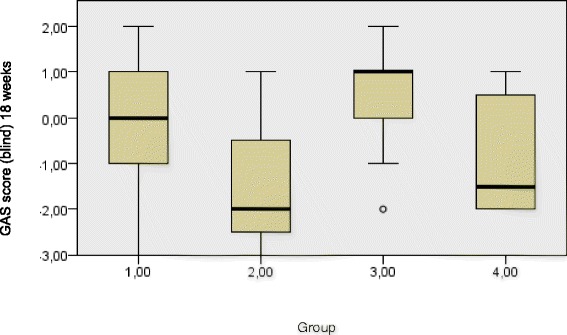
GAS blind score at 18 weeks medians and interquartile ranges per treatment group. Group 1 = BoNT-A + BITT, 2 = BoNT-A-only, 3 = BITT-only, 4 = control. Kruskal Wallis test p 0.059

In Figs. 2 and 3 the medians and ranges of the GAS score per treatment group at 12 and 18 weeks are given. We did not compute the GAS T score, as the GAS, which was scored blindly on a video, was only performed at one treatment goal, so this had no statistical implications [25]. The BITT-only group performed significantly better at 12 weeks compared to the other groups. In the BITT-only group, the GAS score improved from −2 (baseline) to 0.50 (median; interquartile range, IQR 0 – 2), this is a significant difference. At 18 weeks the GAS improved to 1 (IQR −0.25 – 1.25) and at 24 weeks the GAS was 0 (IQR −0.25 – 1.25). The BoNT-A + BITT group also improved, but to a lesser extent: at 12 weeks to 0 (IQR −0.75 – 1.75), at 18 weeks to 0 (IQR −0.75 – 1.50) and at 24 weeks to 0 (IQR −1.0 – 0). The BoNT-A-only group scored worse at all measurement sessions.

## Discussion

In this study, which was set up as an RCT with a factorial design, we investigated the effect of BoNT-A in the upper extremity and/or BITT on bimanual performance, measured with the AHA and the AK questionnaire; on the amount and quality of use of the affected limb in standardized, age appropriate, bimanual tasks, measured with the OSAS; and on goal achievement, measured with the COPM and the, by video observation blinded, GAS score. Due to disappointing participant enrolment, the design had to be adapted to the parents’ preference for either BoNT-A + BITT or just the BITT-only programme. Limitations of this study are the small numbers with unequal group sizes. Therefore, the original RCT became a clinical study. Nevertheless consistent results were found.

Although the differences between the groups were not significant, the children in the BITT group (and control group with younger children) showed a positive effect on the AHA score, which was our primary outcome measurement, at 12 weeks. BITT has a significant positive effect on bimanual performance, measured with the AK and on goal achievement, measured with the COPM and the blinded GAS score, not only at 12 weeks, the end of the therapy programme, but also later at 18 and 24 weeks. Task specific exercise clearly leads to achievement of the goals set by the children themselves and, to a lesser extent, to improvement in bimanual performance. Improvement of functional unimanual and bimanual grip strength by BITT [17] possibly supports this.

In our study BoNT-A seems to have a negative effect on bimanual performance, measured with the AHA. The no-BoNT-A and the BITT-only group AHA score increased from 0 to 12 weeks, whereas the BoNT-A and the BoNT-A + BITT AHA score did not. This is in contradiction with the results of Ferrari et al., who found a significant increase of the AHA raw scores at 3 months in the BoNT-A group (*n* = 11) in a placebo controlled trial of 27 children with uCP, aged 3 to 12 years old, who all received goal directed therapy [26]. They stated that children with the House functional score [27] 4 and 5 would possibly benefit most from BoNT-A combined with goal directed therapy. These children are comparable with the children in our study. At 6 months both groups showed equal improvement on the AHA in their study. Another recent RCT investigating the effects of repeated BoNT-A injections combined with OT and splinting compared with OT alone in very young children with uCP (median age 3y 1 month) showed that 6 out of 10 children of the BoNT-A/OT group (*n* = 10) improved ≥ 5u on the AHA at 12 months compared with 1 out of 10 in the OT group (*n* = 10) [28]. This study concerns very young children with a lower House functional score (1 – 3) but a similar degree of severity of impairment according to the Zancolli grade. Olesch et al. who studied also very young children, did not found a positive effect of additional BoNT-A on the activity level, measured with the QUEST, but they found positive effects on the GAS [7].

In our study, BoNT-A had a negative effect in achieving the first COPM goal and at the GAS score. In the study of Ferrari et al.[26] goal achievement with the GAS was significantly better in the BoNT-A group, whereas in the study of Lidman et al. [28] there was no significant difference between the study groups in improvement on the COPM.

With the OSAS we found that BoNT-A has a positive effect on the amount of use of the affected hand; especially during the time it works at 6 weeks in the sandwich-making task, but also later on in the beading task in the younger age group. The grasp finger score in the BoNT-A group of the sandwich-making task showed significantly more improvement at 6 weeks when comparing BoNT-A to no-BoNT-A. This is consistent with the increase in the amount of use in this task at 6 weeks and the fact that BoNT-A was injected in the thumb adductor, leading to improved thumb abduction, which is necessary for this task. BoNT-A injections in the wrist and finger flexors led to a significantly improved wrist position in the quality of grasp and hold wrist scores in several tasks, due to the effect of tone reduction of the wrist and finger flexors, during the working time at 6 and 12 weeks in the older age group. In the younger children this effect lasted even longer. Given that the known smallest detectable differences of the quality of grasp and hold wrist scores in these tasks vary from 0.34 to 0.87, the found differences are substantial [18].

Our OSAS results of improvement of wrist position and thumb abduction after BoNT-A are in agreement with the positive effect of BoNT-A and OT, compared to OT alone, found in two reviews [1, 8] at 3 months on the MUUL and the QUEST, as these instruments measure also quality of movement, wrist position and fluency.

Improvement at the GAS score by BoNT-A + OT was also reported in these reviews and in the study of Ferrari et al. [26] The GAS T-score of several goals used in the studies in the reviews was not blinded. This may have biased their results because in contrast, in our study a negative additional effect of BoNT-A on the GAS score was found. Here, trained OTsts scored the GAS on a videotape of the main goal; unaware at which measurement session the videotape was taken. Although in this study the children who received BoNT-A improved also at the COPM, the improvement was clearly less than in the children who did not receive BoNT-A or those who received BITT-only. As the study of Ferrari et al. [26] was a placebo controlled RCT, their positive findings on the GAS of BoNT-A speak against our results. However, they discussed the importance of the individualised goal directed therapy and goal setting, because also the placebo group achieved their goals.

## Conlusions

In conclusion, BoNT-A has a positive effect on the quality of movement and amount of use of the affected UE especially during the expected working time and somewhat longer in younger children. Contrary to other studies, we found no additional effect of BoNT-A on bimanual performance and goal achievement. BITT on the other hand, has a clear positive effect on goal achievement and bimanual performance. To improve bimanual performance and goal achievement in children with spastic uCP who are able to actively open their hand, intensive task specific bimanual training is, in our opinion, the first choice of treatment. In children aged 6 years or younger with severe spasticity of the affected hand leading to limited functional hand use, additional BoNT-A injections are to be considered.

## Additional files

Additional file 1:
**AHA unit-scores per child and BoNT-A dose in units per child.** (DOCX 18 kb) Additional file 2:
**Characteristics and outcome measures at baseline younger age group 2.5–6 years old.** (DOC 48 kb) Additional file 3:
**Characteristics and outcome measures at baseline older age group 7–12 years old.** (DOC 53 kb)
