# Supplementary material for: Effects of botulinum toxin A and/or bimanual task-oriented therapy on upper extremity activities in unilateral Cerebral Palsy: a clinical trial
Source: BMC Neurol. 2015 Aug 19;15:143. doi: 10.1186/s12883-015-0404-3 (PMC4544795; doi:10.1186/s12883-015-0404-3)
Supplement: Additional file 1: — AHA unit-scores per child and BoNT-A dose in units per child. (DOCX 18 kb) [file 12883_2015_404_MOESM1_ESM.docx]

Additional file 1. AHA unit-scores per child and BoNT-A dose in units per child.

| number | age | group | Zancolli | AHAut2 | AHAut4 | AHAut6 | AP | FPB | FCU | FCR | PT | FDP | FDS | BR | BB | FPL |
| --- | --- | --- | --- | --- | --- | --- | --- | --- | --- | --- | --- | --- | --- | --- | --- | --- |
| Vo41 | 8 | 2 | 2 | 65 | 62 | 59 | 25 |  | 100 | 150 | 100 |  |  |  |  |  |
| Vj47 | 3 | 2 | 2 | 37 | 38 | 41 | 25 | 25 |  |  | 75 |  | 75 |  |  | 50 |
| Vo50 | 11 | 2 | 1 | 63 | 66 | 62 | 25 |  | 100 | 100 | 50 |  |  |  |  |  |
| Ao03 | 8 | 2 | 1 | 52 | 52 | 60 | 50 |  | 100 |  | 50 |  |  | 100 | 100 |  |
| Vj53 | 3 | 2 | 2 | 65 | 66 | 65 | 25 |  | 100 | 75 |  |  |  |  |  |  |
|  |  |  |  |  |  |  |  |  |  |  |  |  |  |  |  |  |
| Vo43 | 11 | 1 | 1 | 64 | 66 | 60 | 25 |  | 100 | 150 | 150 |  |  |  |  |  |
| Vj46 | 4 | 1 | 2 | 48 | 49 | 999 | 25 | 25 | 75 |  | 50 | 75 |  | 50 |  |  |
| Vo49 | 7 | 1 | 1 | 59 | 48 | 52 | 25 |  | 100 |  |  |  |  |  |  |  |
| Aj04 | 4 | 1 | 1 | 42 | 50 | 60 | 25 | 25 |  |  | 75 |  |  | 50 | 100 |  |
| Vj57 | 6 | 1 | 1 | 53 | 62 | 57 | 25 | 25 | 75 | 75 |  |  |  |  |  |  |
| Ao05 | 7 | 1 | 1 | 53 | 57 | 58 | 50 | 25 | 75 |  |  |  | 100 |  |  |  |
| No23 | 10 | 1 | 1 | 59 | 58 | 65 | 25 |  | 125 | 75 |  |  |  |  |  |  |
| Vj58 | 5 | 1 | 2 | 59 | 64 | 64 | 25 |  | 75 |  |  | 75 |  |  |  |  |
| Vo59 | 9 | 1 | 2 | 60 | 50 | 58 | 25 | 25 | 75 | 75 | 100 |  |  |  |  |  |
| Vo60 | 10 | 1 | 3 | 38 | 35 | 37 | 25 |  | 150 | 100 | 100 | 100 |  |  |  |  |
| Ao06 | 12 | 1 | 1 | 69 | 74 | 71 | 50 |  | 100 |  |  |  |  |  |  |  |
| Ao07 | 7 | 1 | 1 | 59 | 60 | 59 | 50 |  | 50 | 50 |  |  |  | 50 | 50 |  |
| Vo66 | 10 | 1 | 1 | 65 | 64 | 64 | 50 |  | 75 | 75 | 100 |  |  |  |  |  |

| number | age | group | Zan colli | AHAut2 | AHAut4 | AHAut6  Number = childnumber.  Group = treatment group: 1 = BoNT-A+BITT, 2 = BoNT-A-only, 3 = BITT-only, 4 = control.  Zancolli grade 1 = I, 2 = IIA, 3 = IIB.  AHAu = AHA units t2=baseline, t4=12 weeks, t6=24 weeks. 999 = missing values.  AP = adductor pollicis, FPB = flexor pollicis brevis, FCU = flexor carpi ulnaris, FCR = flexor carpi radialis, PT = pronator teres, FDP = flexor digitorum profundus, FDS = flexor digitorum superficialis, BR = brachioradialis, BB = biceps brachii, FPL = flexor pollicis longus.  Significant improvement with Wilcoxon signed rank test in AHA unit-score from baseline to 12 weeks within group in the BITT group (BoNT-A +BITT and BITT-only), in the no-BoNT-A group (BITT-only and control), and in the BITT-only group. There were no significant differences between the groups. |
| --- | --- | --- | --- | --- | --- | --- |
| vo44 | 8 | 3 | 2 | 54 | 54 | 53 |
| vj48 | 6 | 3 | 2 | 49 | 50 | 47 |
| Vj51 | 5 | 3 | 1 | 75 | 78 | 76 |
| Vo52 | 10 | 3 | 2 | 59 | 62 | 54 |
| Ao2 | 7 | 3 | 1 | 54 | 60 | 59 |
| vo54 | 7 | 3 | 1 | 62 | 76 | 62 |
| nj21 | 4 | 3 | 1 | 60 | 66 | 59 |
| no22 | 7 | 3 | 2 | 59 | 60 | 60 |
| vo61 | 11 | 3 | 1 | 62 | 62 | 63 |
| vj62 | 5 | 3 | 1 | 83 | 83 | 81 |
| vo63 | 11 | 3 | 3 | 35 | 39 | 35 |
|  |  |  |  |  |  |  |
| vo42 | 7 | 4 | 1 | 57 | 58 | 64 |
| vj45 | 5 | 4 | 2 | 52 | 52 | 999 |
| aj01 | 5 | 4 | 3 | 59 | 60 | 60 |
| vo55 | 9 | 4 | 3 | 30 | 41 | 999 |
| vj56 | 4 | 4 | 2 | 49 | 50 | 999 |
| vj64 | 4 | 4 | 1 | 50 | 53 | 67 |
