# Supplementary material for: Effects of botulinum toxin A and/or bimanual task-oriented therapy on upper extremity activities in unilateral Cerebral Palsy: a clinical trial
Source: BMC Neurol. 2015 Aug 19;15:143. doi: 10.1186/s12883-015-0404-3 (PMC4544795; doi:10.1186/s12883-015-0404-3)
Supplement: Additional file 2: — Characteristics and outcome measures at baseline younger age group 2.5–6 years old. (DOC 48 kb) [file 12883_2015_404_MOESM2_ESM.doc]

Additional file 2. Characteristics and outcome measures at baseline younger age group 2.5-6 years old.

| **Younger age group** | **BoNT-A + BITT** | **BoNT-A-only** | **BITT-only** | **control** | **all** |
| --- | --- | --- | --- | --- | --- |
|  | n = 4 | n = 2 | n = 4 | n = 4 | n = 14 |
| **Age** (years) mean (sd) | 4.8 (0.96) | 3.0 (0) | 5.0 (0.82) | 4.5 (0.58) | 4.5 (0.94) |
| **Hemi side** |  |  |  |  |  |
| Hemi right n (%) | 3 (75) | 2 (100) | 2 (50) | 1 (25) | 8 (57.1) |
| Hemi left n (%) | 1 (25) | 0 | 2 (50) | 3 (75) | 6 (42.9) |
| **Zancolli grade** |  |  |  |  |  |
| Zancolli I n (%) | 2 (50) | 0 | 3 (75) | 1 (25) | 6 (28.6) |
| Zancolli IIA n (%) | 2 (50) | 2 (100) | 1 (25) | 2 (50) | 7 (33.3) |
| Zancolli II B n (%) | 0 | 0 | 0 | 1 (25) | 1 (4.8) |
| **MACS** |  |  |  |  |  |
| MACS I n (%) | 1 (25) | 1 (50) | 2 (50) | 2 (50) | 6 (42.9) |
| MACS II n (%) | 1 (25) | 0 | 2 (50) | 2 (50) | 5 (35.7) |
| MACS III n (%) | 2 (50) | 1 (50) | 0 | 0 | 3 (14.3) |
| **AHA** |  |  |  |  |  |
| Units mean (sd) | 50.5 (7.2) | 51.0 (19.8) | 66.8 (15.2) | 52.5 (4.5) | 55.8 (12.4) |
| **ABILHAND-Kids** |  |  |  |  |  |
| Logit units mean (sd) | 0.147 (0.517) | 1.475 (0.221) | 1.225 (0.603) | 0.408 (1.423) | 0.719 (0.956) |
| **COPM** mean of 3 goals |  |  |  |  |  |
| Performance mean (sd) | 4.0 (1.6) | 4.7 (0.5) | 3.8 (1.8) | 3.3 (1.8) | 3.8 (1.5) |
| Satisfaction mean (sd) | 4.4 (1.9) | 5.8 (1.2) | 4.6 (1.5) | 3.7 (2.2) | 4.5 (1.8) |
| **OSAS percentage of use** treading beads mean (sd) | 76.7 (10.3) | 59.8 (30.4) | 84.5 (9.9) | 82.0 (8.3) | 78.0 (14.3) |
| **OSAS quality of use** younger children |  |  |  |  |  |
| **Grasp fingers** treading beads mean (sd) | 1.699 (0.250) | 1.385 (0.545) | 1.793 (0.226) | 1.595 (0.232) | 1.651 (0.283) |
| **Grasp wrist** Treading beads mean (sd) | 2.158 (0.530) | 2.215 (0.834) | 2.489 (0.959) | 1.926 (0.761) | 2.194 (0.717) |
| **Grasp wrist** Pop-Onz mean (sd) | 2.104 (0.790) | 2.705 (0.537) | 2.413 (0.798) | 1.849 (0.663) | 2.205 (0.713) |
| **Hold wrist** Treading beads mean (sd) | 2.225 (0.706) | 2.223 (0.732) | 2.653 (0.992) | 2.311 (0.920) | 2.371 (0.784) |
| **Hold wrist**  Pop-Onz mean (sd) | 2.073 (0.679) | 2.465 (0.488) | 2.630 (0.909) | 1.965 (0.658) | 2.257 (0.708) |
