# Supplementary material for: Effects of botulinum toxin A and/or bimanual task-oriented therapy on upper extremity activities in unilateral Cerebral Palsy: a clinical trial
Source: BMC Neurol. 2015 Aug 19;15:143. doi: 10.1186/s12883-015-0404-3 (PMC4544795; doi:10.1186/s12883-015-0404-3)
Supplement: Additional file 3: — Characteristics and outcome measures at baseline older age group 7–12 years old. (DOC 53 kb) [file 12883_2015_404_MOESM3_ESM.doc]

Additional file 3. Characteristics and outcome measures at baseline older age group 7-12 years old.

| **Older age group** | **BoNT-A + BITT** | **BoNT-A-only** | **BITT-only** | **control** | **all** |
| --- | --- | --- | --- | --- | --- |
|  | n = 9 | n = 3 | n = 7 | n = 2 | n = 21 |
| **Age** (years) mean (sd) | 9.2 (1.9) | 9.0 (1.7) | 8.7 (1.9) | 8 (1.4) | 8.9 (1.7) |
| **Hemi side** |  |  |  |  |  |
| Hemi right n (%) | 6 (66.7) | 1 (33.3) | 4 (57.1) | 0 | 11 (52.4) |
| Hemi left n (%) | 3 (33.3) | 2 (66.7) | 3 (42.9) | 2 (100) | 10 (47.6) |
| **Zancolli grade** |  |  |  |  |  |
| Zancolli I n (%) | 7 (77.8) | 2 (66.7) | 4 (57.1) | 1 (50.0) | 14 (48.3) |
| Zancolli IIA n (%) | 1 (11.1) | 1 (33.3) | 2 (28.6) | 0 | 4 (13.8) |
| Zancolli II B n (%) | 1 (11.1) | 0 | 1 (14.3) | 1 (50.0) | 3 (10.3) |
| **MACS** |  |  |  |  |  |
| MACS I n (%) | 1 (1.11) | 1 (33.3) | 2 (28.6) | 1 (50.0) | 5 (17.2) |
| MACS II n (%) | 6 (66.7) | 1 (33.3) | 2 (28.6) | 1 (50.0) | 10 (34.5) |
| MACS III n (%) | 2 (22.2) | 1 (33.3) | 3 (42.9) | 0 | 6 (20.7) |
| **AHA** |  |  |  |  |  |
| units mean (sd) | 58.4 (8.9) | 60.0 (7.0) | 55.0 (9.4) | 43.5 (19.1) | 56.1 (10.1) |
| **ABILHAND-Kids** |  |  |  |  |  |
| Logit units mean (sd) | 1.675 (0.588) | 1.338 (0.578) | 1.528 (0.868) | 1.702 (1.686) | 1.580 (0.746) |
| **COPM** mean of 3 goals |  |  |  |  |  |
| Performance mean (sd) | 4.2 (1.6) | 2.7 (0.3) | 3.2 (1.1) | 3.0 (0.5) | 3.5 (1.4) |
| Satisfaction mean (sd) | 5.1 (2.3) | 4.3 (2.3) | 3.8 (1.6) | 4.5 (0.7) | 4.5 (1.9) |
| **OSAS percentage of use** sandwich-making mean (sd) | 76.2 (10.5) | 80.5 (8.7) | 78.5 (8.9) | 81.3 (3.3) | 78.1 (8.9) |
| **OSAS quality of use** older children |  |  |  |  |  |
| **Grasp fingers** Sandwich-making mean (sd) | 1.785 (0.394) | 1.923 (0.125) | 1.757 (0.310) | 1.498 (0.506) | 1.768 (0.341) |
| **Grasp wrist** Sandwich-making mean (sd) | 1.653 (0.691) | 1.337 (0.292) | 1.564 (0.791) | 2.125 (0.587) | 1.623 (0.666) |
| **Grasp wrist** Construction small mean (sd) | 1.927 (0.885) | 1.425 (0.383) | 1.446 (0.766) | 2.333 (0.944) | 1.734 (0.803) |
| **Grasp wrist** Construction large mean (sd) | 1.515 (0.581) | 1.272 (0.177) | 1.413 (0.719) | 1.890 (0.629) | 1.482 (0.582) |
| **Hold fingers** Sandwich-making mean (sd) | 2.032 (0.402) | 2.260 (0.310) | 1.829 (0.447) | 1.568 (0.774) | 1.953 ( 0.449) |
| **Hold fingers** Construction small mean (sd) | 2.139 (0.211) | 2.005 (0.130) | 1.988 (0.539) | 2.056 (1.315) | 2.061 (0.445) |
| **Hold fingers** Construction large mean (sd) | 2.308 (0.255) | 2.213 (0.470) | 2.091 (0.532) | 1.973 (0.824) | 2.190 (0.426) |
| **Hold wrist** Sandwich-making mean (sd) | 1.725 (0.620) | 1.397 (0.524) | 1.722 (0.875) | 2.265 (0.028) | 1.729 (0.675) |
| **Hold wrist** Construction small mean (sd) | 1.930 (0.945) | 1.161 (0.579) | 1.764 (0.824) | 2.485 (0.686) | 1.882 (0.819) |
| **Hold wrist** Construction large mean (sd) | 1.689 (0.714) | 1.687 (0.671) | 1.491 (0.720) | 1.328 (0.173) | 1.588 (0.650) |
